# Testing a German Adaption of the Entrapment Scale and Assessing the Relation to Depression

**DOI:** 10.1155/2010/501782

**Published:** 2010-11-04

**Authors:** Manuel Trachsel, Tobias Krieger, Paul Gilbert, Martin Grosse Holtforth

**Affiliations:** ^1^Department of Psychology, University of Bern, Switzerland; ^2^Department of Psychology, University of Zurich, Binzmuehlestraße 14/Box 19, 8050 Zurich, Switzerland; ^3^Kingsway Hospital, Mental Health Research Unit, University of Derby, Derby, Derbyshire DE22 3LZ, UK

## Abstract

The construct of *entrapment* is used in evolutionary theory to explain the etiology of depression. The perception of entrapment can emerge when defeated individuals want to escape but are incapable. Studies have shown relationships of entrapment to depression, and suicidal tendencies. The aim of this study was a psychometric evaluation and validation of the Entrapment Scale in German (ES-D). 540 normal subjects completed the ES-D along with other measures of depressive symptoms, hopelessness, and distress. Good reliability and validity of the ES-D was demonstrated. Further, whereas entrapment originally has been regarded as a two-dimensional construct, our analyses supported a single-factor model. Entrapment explained variance in depressive symptoms beyond that explained by stress and hopelessness supporting the relevance of the construct for depression research. These findings are discussed with regard to their theoretical implications as well as to the future use of the entrapment scale in clinical research and practice.

## 1. Introduction

Assuming a certain degree of adaptivity of behavior and emotion, evolutionary theorists have suggested various functions of moodiness and depression. Whereas adaptive mechanisms may become functionally maladaptive [1, 2], there have been many attempts to explain potentially adaptive functions of depression. For example, Price [3] suggested that depression evolved from the strategic importance of having a de-escalating or losing strategy. Social rank theory [4, 5] built on this and suggests that some aspects of depression, such as mood and drive variations, may have evolved as mechanisms for regulating behavior in contexts of conflicts and competition for resources and mates. Hence, subordinates are sensitive to down rank threats and are less confident than dominants, while those who are defeated will seek to avoid those who defeated them. Depression may also serve the function to help individuals disengage from unattainable goals and deal with losses [6]. 

Social rank theory (e.g., [4]) links defeat states to depression. Drawing on Dixon's arrested defences model of mood variation [7, 8], this theory suggests that especially when stresses associated with social defeats and social threats arise, individuals are automatically orientated to fight, flight or both. Usually, either of those defensive behaviors will work. So, flight and escape remove the individual from the conditions in which stress is arising (e.g., threats from a dominant), or anger/aggression curtails the threat. These defensive behaviors typically work for nonhuman animals. However, for humans, such basic fight and flight strategies may be less effective facing the relatively novel problems of living in modern societies, perhaps explaining the prevalence of disorders such as depression [8]. Dixon suggested that in depression, defensive behaviors can be highly aroused but also blocked and arrested and in this situation depression ensues. Dixon et al. [8] called this *arrested flight*. For example, in lizards, being defeated but able to escape has proven to be less problematic than being defeated and being trapped. Those who are in caged conditions, where escape is impossible, are at risk of depression and even death [9]. Gilbert [4, 10] and Gilbert and Allan [5] noted that depressed individuals commonly verbalize strong escape wishes and that feelings of entrapment and desires to escape have also been strongly linked to suicide, according to O'Connor [11]. In addition they may also have strong feelings of anger or resentment that they find difficult to express or become frightening to them.

Gilbert [4] and Gilbert and Allan [5] proposed that a variety of situations (not just interpersonal conflicts) that produce feeling of defeat, or uncontrollable stress, which stimulate strong escape desires but also makes it impossible for an individual to escape, lead the individual to a perception of *entrapment*. They defined entrapment as a desire to escape from the current situation in combination with the perception that all possibilities to overcome a given situation are blocked. Thus, theoretically entrapment follows defeat if the individual is not able to escape. This inability may be due to a dominant subject who does not offer propitiatory gestures following antagonistic competition, or if the individual keeps being attacked.

In contrast to individuals who feel helpless (cf. the concept of learned helplessness [12]), which focus on perceptions of control, the entrapped model focuses on the outputs of the threat system emanating from areas such as the amygdala [13]. In addition, depressed people are still highly motivated and would like to change their situation or mood state. It was also argued that, unlike helplessness, entrapment takes into account the social forces that lead to depressive symptoms, which is important for group-living species with dominance hierarchies such as human beings [14]. Empirical findings by Holden and Fekken [15] support this assumption. Gilbert [4] argued that the construct of entrapment may explain the etiology of depression better than learned helplessness, because according to the theory of learned helplessness, helpless individuals have already lost their flight motivation whereas entrapped individuals have not.

According to Gilbert [4], the perception of entrapment can be triggered, increased, and maintained by external factors but also internal processes such as intrusive, unwanted thoughts and ruminations can play an important role (e.g., [16, 17]). For example, ruminating on the sense of defeat or inferiority may act as an internal signal of down-rank attack that makes an individual feel increasingly inferior and defeated. Such rumination may occur despite the fact that an individual successfully escaped from an entrapping external situation because of feelings of failure, which may cause a feeling of internal entrapment. For example, Sturman and Mongrain [18] found that internal entrapment increased following an athletic defeat. Moreover, thoughts and feelings like “internal dominants” in self-critics may exist that can also activate defensive behaviors.

For the empirical assessment of entrapment, Gilbert and Allan [5] developed the self-report *Entrapment Scale* (ES) and demonstrated its reliability. Using the ES, several studies have shown that the perception of entrapment is strongly related to low mood, anhedonia, and depression [5, 19–21]. Sturman and Mongrain [22] found that entrapment was a significant predictor of recurrence of major depression. Further, Allan and Gilbert [23] found that entrapment relates to increased feelings of anger and to a lower expression of these feelings. In a study by Martin et al. [24], the perception of entrapment was associated with feelings of shame, but not with feelings of guilt. Investigating the temporal connection between depression and entrapment, Goldstein and Willner [25, 26] concluded that the relation between depression and entrapment is equivocal and might be bilateral; that is, entrapment may lead to depression and vice versa. 

Entrapment was further used as a construct explaining suicidal tendency. In their *cry-of pain-model*, Williams and Pollock [27, 28] argued that suicidal behavior should be seen as a cry of pain rather than as a cry for help. Consistent with the concept of arrested flight, they proposed that suicidal behavior is reactive. In their model, the response (the cry) to a situation is supposed to have the following three components: defeat, no escape potential, and no rescue. O'Connor [11] provided empirical support in a case control study by comparing suicidal patients and matched hospital controls on measures of affect, stress, and posttraumatic stress. The authors hypothesized that the copresence of all three cry-of-pain variables primes an individual for suicidal behavior. The suicidal patients, with respect to a recent stressful event, reported significantly higher levels of defeat, lower levels of escape potential, and lower levels of rescue than the controls. Furthermore, Rasmussen et al. [21] showed that entrapment strongly mediated the relationship between defeat and suicidal ideation in a sample of first-time and repeated self-harming patients. Nevertheless, there has also been some criticism of the concept of entrapment as it is derived from animal literature [29].

To our knowledge so far, there is no data on the retest reliability or the temporal stability of the Entrapment Scale. Because entrapment is seen as a state-like rather than a trait-like construct, its stability is likely dependent on the stability of its causes. Therefore, if the causes of entrapment are stable (e.g., a long-lasting abusive relationship), then also entrapment will remain stable over time. In contrast, for the *Beck Hopelessness Scale* (BHS), there are studies assessing temporal stability that have yielded stable trait-like components of hopelessness [30]. Young and coworkers [30] stated that the high stability of hopelessness is a crucial predictor of depressive relapses and suicidal attempts. For the *Perceived Stress Questionnaire* (PSQ), there are studies examining retest reliability. The PSQ has shown high retest reliability over 13 days (*r* = .80) in a Spanish sample [31]. It is to be expected that with longer retest intervals as in the present study (3 months), the stability of perceived stress will be substantially lower. We, therefore, expect the stability of entrapment to be higher than that of perceived stress as a state-like construct, but lower than that of hopelessness, which has been shown to be more trait-like [32].

Previous research is equivocal regarding the dimensionality of the entrapment construct. Internal and external entrapment were originally conceived as two separate constructs (cf. [5]) and were widely assessed using two subscales measuring entrapment caused by situations and other people (e.g., “*I feel trapped by other people*”) or by one's own limitations (e.g., “*I want to get away from myself*”). The scores of the two subscales were averaged to result in a total entrapment score in many studies. However as Taylor et al. [33] have shown, entrapment may be best conceptualized as a unidimensional construct. This reasoning is supported by the observation that some of the items of the ES cannot easily be classified either as internal or external entrapment and because the corresponding subscales lack face validity (e.g., “*I am in a situation I feel trapped in*” or “*I can see no way out of my current situation*”).

## 2. Aim of the Present Study

Empirical evidence indicates that entrapment can be reliably assessed using the ES. Furthermore, the perception of entrapment seems to be a frequent experience of depressed persons. The aims of the present study were (a) to develop a German version of the ES, (b) to test its factorial structure, (c) to demonstrate construct validity of the German version of the ES by replicating previously found associations with other constructs, (d) to support the distinctness of the entrapment construct for the explanation of depressive symptoms, and (e) to investigate the stability of the ES.

Thus, the following hypotheses were tested in the present study.

1. The ES-D demonstrates high internal consistency.
2. The ES shows a single-factor structure.
3. The German version of the ES demonstrates acceptable construct validity. This means that entrapment measured with the ES-D positively correlates with depressive symptoms, with perceived general stress level, and with hopelessness.
4. Entrapment shows incremental validity by explaining a substantial extra proportion of the variance in depressive symptoms, after controlling for perceived stress and hopelessness.
5. Entrapment is less stable than hopelessness but more stable than perceived stress.

## 3. Materials and Method

### 3.1. Participants

The study included three samples that involved a total of 540 participants. The samples were a sample of subjects completing a paper-pencil version of the ES (PP, *N* = 170) and a sample that completed the ES online (OL, *N* = 370). The third sample was a subset of 100 subjects from the OL sample participating in the retest assessment. After three months, all subjects of the OL sample were invited once again to participate in the retest assessment whereof 100 subjects (27%) followed the invitation. Two-hundred and seventy subjects (73%) did not take part in the retest assessment without indication of reasons.

Because of the different presentation forms (PP versus OL) it was not methodically clear whether these forms would show comparable psychometric properties. Therefore PP and OL were described separately throughout the paper.

Participants were recruited from the social environment of the first two authors and of two research assistants. These four persons wrote emails (for OL samples) and letters (for PP samples) to colleagues and acquaintances and posted links to the online questionnaire in various social internet networks. All participants were volunteers from Germany or German-speaking Switzerland, and they did not receive incentives. All subjects gave their informed consent for the study. 

Table 1 gives an overview of the sociodemographic characteristics of the three samples. Because the participants were recruited from the social environment of the authors and to assure the participants' anonymity, the participants indicated their age by checking an age group instead of providing the precise age. The PP sample consisted of 170 participants (96 females, 74 males; 63.2% female). Fifty-three (31%) participants were between the age of 18 and 26; 64 (38%) were between 26 and 40; 47 (28%) were between 40 and 60; 6 (3%) were older than 60 years. The OL sample consisted of 370 participants (234 females, 136 males; 56.5% female). Participants completed the questionnaires online after receiving an invitation via email. One hundred eleven (30%) of them were between the age of 18 and 26; 207 (56%) between 26 and 40; 33 (9%) were between 40 and 60; 19 (5%) were older than 60 years. After a second invitation by email, a total of 100 participants (27.0%) completed a retest after a period of 3 months.

### 3.2. Measures

#### 3.2.1. Entrapment Scale (ES-D)

The 16 items of the original ES [5] were translated into German by the first author with the permission of the author of the original scale. A bilingual person translated the items back into English, which led to comparable items with small deviations from the original version. Each deviation was examined and corrected in order to optimize the German translation [34]. Items 1 to 10 had originally been generated by Gilbert and Allan [5] to measure *External Entrapment*, and items 11 to 16 were designed to assess *Internal Entrapment* (see Table 3). Participants were asked to indicate the degree to which the items represented their thoughts and feelings during the last week on a 5-point Likert scale (*not at all, a little bit, moderately, quite a bit, *and* extremely*). 

#### 3.2.2. Center for Epidemiologic Studies Depression Scale (CES-D)

The Center for Epidemiologic Studies Depression Scale (CES-D [35]) is a self-report questionnaire that has been developed for studies involving nonclinical samples to assess the presence and duration of depressive affect, motor inhibition, and negative thought patterns. In this study, we used the short German version of the CES-D (Allgemeine Depressions Skala, ADS-K, [36]). The ADS-K consists of 15 items (e.g., “I feel sad”, or “Everything is tiring for me”). The ADS-K showed good internal consistency in clinical and nonclinical samples (*α* = .93 for a depressive sample, *α* = .90 in a general sample; 36).

#### 3.2.3. Beck Hopelessness Scale (BHS)

The Beck Hopelessness Scale (BHS [37]) is a 20-item self-report scale measuring the degree of pessimism about the future (e.g., “In the future I expect to succeed in what concerns me most”, or “I just don't get the breaks and there is no reason to believe I will in the future”). Beck and Steer [38] found for the BHS a high internal consistency as well as correlations with clinical ratings and other hopelessness measures. Krampen [39] translated the BHS into German and presented a short form, for which good reliability and validity estimates are reported. The German form of the BHS exists in two parallel forms with 10 items each. We used parallel form A in this study.

#### 3.2.4. Perceived Stress Questionnaire (PSQ)

The Perceived Stress Questionnaire (PSQ [40]) is a self-report questionnaire that assesses subjectively experienced stress independently of specific and objective triggers [40] (e.g., “You feel that too many demands are being made on you”, or “Your problems seem to be piling up”). In this study, a German revised version of the PSQ [41] was used, which consists of 20 items scored on a 4-point Likert-scale format (*almost never*, *sometimes*, *often*, and *mostly*). The PSQ has shown good scale properties and validity in English-, German- and Italian-speaking samples.

### 3.3. Data Analysis

Data analysis proceeded in three steps using the PASW Statistics 18.0 software. First, differences between samples were investigated by *t*-tests and *χ* ^2^-tests. The goals were to compare the two samples that used different answering formats (online versus paper-pencil), and to make sure that the retest sample did not differ from the larger OL sample. Secondly, the means, standard deviations, and reliability coefficients (Cronbach's *α* and Spearman-Brown *r*) were calculated, and exploratory factor analyses (EFA) were conducted. On theoretical grounds, we expected that the factors were correlated, so that an oblique rotation was chosen. We computed the Pearson correlations to establish construct validity and hierarchical multiple regression analyses to test incremental validity. Third, we computed paired-samples *t*-tests and intraclass correlations (ICC) in the retested sample to investigate sensitivity and stability over time. To correct for skewness, variables measuring entrapment and depressive symptoms were log transformed after adding 1 to each subject's score.

## 4. Results

### 4.1. Sample Comparisons

Table 2 gives an overview of means and standard deviations in the samples of our study. Participants of the OL-sample were more depressed (*t*[538] = 7.34, *P* < .01, *d* = .63), felt more entrapped (*t*[538] = 1.98, *P* < .05, *d* = .17), and more stressed (*t*[538] = 2.99, *P* < .01, *d* = .26) than participants of the PP-sample. The two samples did not differ regarding hopelessness (*t*[538] = .95*, n.s.*, *d* = .08). Further, the PP and the OL sample were split according to the cutoff of the CES-D which is considered indicative of dividing subjects with clinically relevant depressive episodes from healthy subjects [42]. The PP (12.9%; *n* = 22) and the OL sample (9.7%; *n* = 36) did not differ regarding the amount of participants exceeding the cutoff (*χ* ^2^ [1, *N* = 540] = 1.25, *P* > .05).

As stated above, the retest-sample (RT) was a subsample of the OL-sample. The 100 retested persons were significantly older than the subjects not taking part in the retest assessment (*χ* ^2^ [3, *N* = 370] = 9.43, *P* < .05, *ϕ*_*C*_ = .16). At baseline, (*t*_1_) subjects taking part at the retest 3 months later (*t*_2_) did not differ from persons not taking part in the retest with respect to depressiveness, perceived stress, or hopelessness, but differed regarding entrapment (*t*[368] = −2.84, *P* < .01, *d* = .30).

### 4.2. Factorial Structure

A principal-axis EFA was conducted for both samples using the covariance matrix and oblimin rotation (*δ* = 0). Data was suitable for an EFA according to Bartlett's test (PP: *χ* ^2^[120] = 2213.27, *P* < .01; OL: *χ* ^2^[120] = 4339.90, *P* < .01) and Kaiser's measure of sampling adequacy [MSA, 43; PP: AICs between .89–.97; OL: AICs between .87–.97], so that the items could be considered apt for factor analyses. The Kaiser-Meyer-Olkin measure (KMO) also indicated that sample size was adequate in both samples (KMO_PP_ = .94; KMO_OL_ = .95). 

Following the study of Taylor et al. [33], the algorithm extracting factors were based upon parallel analysis [43, 44]. Parallel analysis is a method to identify the optimal number of factors to extract based on the number of factors with Eigenvalues exceeding those expected by chance [45]. The chance values are derived from randomly generated datasets. The parallel analysis was conducted using an PASW syntax provided by O'Connor [45]. Results of the two parallel analyses indicate that only the first Eigenvalue for both real datasets (first five Eigenvalues in the real datasets: PP: 10.51, 1.21, .84, .71, .67; OL: 10.66, 1.36, .90, .76, .66) exceeded those in each case derived from 10,000 randomly generated datasets by chance (95%). Therefore, a single-factor solution was supported in both datasets. The standardized factor loadings of the single-factor model for both samples are shown in Table 3. For the paper-and-pencil sample (*N* = 170), the single-factor solution explained 60.72 % of the variance. For the online sample (*N* = 370), the single-factor solution explained 58.52% of the variance. In both cases, all items correlated significantly (*P* < .01) with the main factor (range: PP: *r* = .68–.86; OL: *r* = .54–.86).

### 4.3. Reliability

 Cronbach's *α* of the ES-D was .95 for both the PP and the OL samples. These values were similar to those found in studies using the original ES. Split-half reliability of the ES-D was *r* = .92 (Spearman-Brown) in both samples.

### 4.4. Construct Validity

 To examine the construct validity of the ES-D, entrapment scores were correlated with total depression scores, with hopelessness scores, and with perceived stress scores in both samples (see Table 4). Entrapment correlated highly significantly and positively with depressive symptoms, stress, and hopelessness. The height of these correlations was comparable with correlations found in studies using the original ES, therefore supporting the validity of the ES-D.

### 4.5. Incremental Validity

 Incremental validity of the ES was operationalized by a significant increment in the explained variance in the prediction of depressive symptoms. The assumptions for multiple linear regressions, including measures for collinearity, were not violated by our data, and no multivariate outliers or influential cases were identified.  Table 5 shows results of the hierarchical regression analyses for the prediction of depressive symptoms in both samples. First, perceived stress and hopelessness were included as predictors (model 1). When entrapment was introduced (model 2), in both regression analyses the feeling of being trapped significantly explained additional variance in depressive symptoms above and beyond that already being explained by stress and hopelessness. Entrapment predicted an additional 10% in the PP sample and an additional 5% in the OL sample. The effect sizes (Cohen's *f* ^2^) for change in Δ*R* ^2^ due to the addition of entrapment were small in both samples (*f*_PP_^2^ = .11, *f*_OL_^2^ = .05).

### 4.6. Sensitivity to Change and Stability

 The sensitivity to change of the ES-D was assessed by analyzing the data of a retest (RT) after 3 months (*n* = 100). As expected, in the RT-sample mean values for entrapment, depressive symptoms, perceived stress, and hopelessness remained unchanged from *t*_1_ to *t*_2_ (all *P*-values of the paired samples *t*-tests >.05). In terms of stability, moderate correlations between values at *t*_1_ and *t*_2_ were found for entrapment (ICC = .67, 95% CI: .55 − .77), perceived stress (ICC = .52, 95% CI: 36 − .65), and depressive symptoms (ICC = .62, .95% CI: 49 − .73) whereas hopelessness showed a comparably higher retest correlation (ICC = .82, CI: .75–.88). When comparing the correlations [46] only the differences between hopelessness and all other constructs were significant.

## 5. Discussion

The entrapment construct embeds depressiveness theoretically into an evolutionary context. The situation of arrested flight or blocked escape, in which a defeated individual is incapable of escaping despite a maintained motivation to escape, may lead to the perception of *entrapment *in affected individuals [8]. In this study, the Entrapment Scale (ES) was translated to German (ES-D), tested psychometrically, and validated by associations with other measures. This study provides evidence that the ES-D is a reliable self-report measure of entrapment demonstrating high internal consistency. The study also shows that the ES-D is a valid measure that relates to other similar constructs like hopelessness, depressive symptoms or perceived stress. Levels of entrapment as measured with the ES-D were associated with depressiveness, perceived stress, and hopelessness, showing moderate to high correlations. Results were consistent with those obtained by Gilbert and Allan [5]. Entrapment explained additional variance in depressiveness beyond that explained by stress and hopelessness. Taken together, the present data support the conception of entrapment as a relevant and distinct construct in the explanation of depression.

The results of our study confirm the findings of Taylor et al. [33], thereby showing that entrapment is only theoretically, but not empirically, separable into internal and external sources of entrapment. The authors even went further by showing that entrapment and defeat could represent a single construct. Although in this study the defeat scale [5] was not included, the results are in line with the assumption of Taylor et al. [33] and support other studies using entrapment a priori as a single construct. However, although this study supports the general idea that escape motivation affects both internal and external events and depression, clinically it can be very important to distinguish between them. For example, in studies of psychosis entrapment can be very focused on internal stimuli, particularly voices [47].

The state conceptualization of entrapment implies that the perception of entrapment may change over time. Therefore, we did not expect retest correlations as high as retest correlations for more trait-like constructs like hopelessness [32]. Since the correlation over time is generally a function of both the reliability of the measure and the stability of the construct, high reliability is a necessary condition for high stability [48]. In this study, we showed that the ES-D is a reliable scale, and we considered retest correlations as an indicator for stability. The intraclass correlation of .67 suggests that entrapment is more sensitive to change than hopelessness (*r* = .82). Furthermore, the state of entrapment seems to be more stable than perceived stress, which may be influenced to a greater extent by external factors. Given the confirmed reliability and validity of the ES-D in this study, we therefore cautiously conclude that entrapment lies between hopelessness and perceived stress regarding stability.

Whereas the high correlation between entrapment and depressive symptoms in this study may be interpreted as evidence of conceptual equivalence, an examination of the item wordings of two scales clearly suggest that these questionnaires assess distinct constructs. However, the causal direction of this bivariate relation is not clear. Theoretically, both directions are plausible. Entrapment may be a cause or a consequence of depressive symptoms, or even both. Unfortunately, studies examining the temporal precedence so far have yielded equivocal results and have methodological shortcomings (e.g., no clinical samples, only mild and transitory depression and entrapment scores with musical mood induction) in order to answer this question conclusively [25, 26]. It remains unclear whether entrapment only is depression specific. Entrapment might not only be associated with depression, but also with other psychological symptoms, or even psychopathology in general. This interpretation is supported by research showing a relation between distress arising from voices and entrapment in psychotic patients [49, 50]. Furthermore, other studies show the relation between entrapment and depressive symptoms [51–53] and social anxiety and shame [54] in psychosis. The usefulness of entrapment as a construct for explaining psychopathologies in humans has been questioned [29]. Due to the present study, it is now possible to investigate entrapment in psychopathology in the German speaking area.

## 6. Limitations and Future Research

The present study has notable limitations. This study used two nonclinical samples. It was not systematically assessed by clinical interviews, whether any of the participants were suffering from clinically relevant depressive episodes. However, a cutoff that is considered indicative of clinically relevant depression was applied [42]. Future studies will need to test the generalizability of the present findings for clinical samples. So far, it remains unclear whether the amount of explained variance in depressiveness by entrapment beyond that explained by stress and hopelessness would disappear in clinical samples with clinically depressed subjects. Moreover, further validation studies will require a clarification of the depression specificity of the entrapment construct. Furthermore, due to the mainly cross-sectional design of the study, we were unable to investigate the causal relations of entrapment with other constructs. Future longitudinal studies with multiple measurement points are at large, focusing on the question whether entrapment is either a cause or a consequence of depressive symptoms, or even both. Moreover, 73% of the OL sample did not take part in the retest assessment without indication of reasons. This considerably dropout rate may have distorted the results concerning retest reliability and temporal stability.

The assumption that individuals feeling trapped in a situation are still highly motivated to change their situation suggests possible implications for research and practice. First, further research needs to identify groups of people who are especially prone to entrapment (e.g., women and men in abusive relationships, self-critics, or people living in bad socioeconomic circumstances). Second, further research has to investigate to what extent the feeling of being trapped can be changed by psychological interventions. If entrapment can be changed, clinicians may set out to find individual sources of entrapment for each patient. Depending on whether he or she deems these potential sources as changeable or stable, challenging and/or accepting them may be the appropriate intervention strategies to help the patient overcome the feeling of being trapped. Such “entrapment-focused interventions” could be integrated in existing programs for the psychotherapy of depression.

## Figures and Tables

**Table 1:** Sociodemographic characteristics of the different samples.

	Sample					
	PP sample (*N* = 170)		OL sample (*N* = 370)		RT sample (*N* = 100)	
	*n*	%	*n*	%	*n*	%
Sex						
Female	96	63.2	234	56.5	64	64.0
Male	74	36.8	136	43.5	36	36.0

Marital status						
Single	24	14.1	103	27.8	26	26.0
Living with a partner/married	137	80.6	253	68.4	70	70.0
Separated/divorced/widowed	9	5.3	11	3.0	4	4.0
missing	0	0	3	0.8	0	0

Age						
18–26	111	30.0	53	31.2	27	27.0
26–40	207	55.9	64	37.6	54	54.0
40–60	33	8.9	47	27.6	16	16.0
60<	19	5.1	6	3.5	3	3

Employment status						
Student	41	24.1	120	32.4	26	26.0
Employed (part-time)	56	32.9	128	34.6	49	49.0
Employed (full-time)	62	36.5	105	28.4	21	21.0
Unemployed/pensioner	4	2.4	14	3.8	3	3.0
missing	9	4.1	3	0.8	1	1.0

PP: Paper-Pencil-Sample, OL: Online-Sample, RT: Retest-Sample. The RT-Sample was a subsample of the OL-Sample.

**Table 2:** Mean values, standard deviations, and cronbach's alphas of the entrapment scale and other constructs.

	Sample								
	PP (*N* = 170)			OL (*N* = 370)			RT (*N* = 100)		
	M	SD	*α*	M	SD	*α*	M	SD	*α*
Entrapment	10.51	12.82	.95	12.08	12.79	.95	13.51	13.52	.95
Depressive symptoms	8.64	7.41	.84	10.53	5.32	.76	10.79	5.77	.80
Stress	39.56	11.73	.91	42.71	11.16	.92	41.87	11.33	.91
Hopelessness	23.32	6.13	.77	23.88	6.38	.77	23.72	6.82	.80

Scores for all scales are presented in raw form. PP: Paper-Pencil-Sample, OL: Online-Sample, RT = Retest-Sample.

**Table 3:** Standardized factor loadings of the entrapment scale for a single-factor solution.

Item-Nr.	Item	Subscale	PP (*N* = 170)	OL (*N* = 370)
16	I feel I'm in a deep hole I cannot get out of	IE	.87	.74
	(Ich fühle mich in einem tiefen Loch, aus dem ich nicht hinaus kann)			
7	I can see no way out of my current situation	EE	.85	.82
	(Ich kann keinen Weg aus meiner momentanen Situation sehen)			
11	I want to get away from myself	IE	.84	.82
	(Ich würde gerne vor mir selbst flüchten)			
9	I have a strong desire to get away and stay away from where I am now	EE	.84	.85
	(Ich habe den starken Wunsch, meine momentane Situation zu verlassen und von ihr fernzubleiben)			
14	I feel trapped inside myself	IE	.83	.81
	(Ich fühle mich in mir selbst gefangen)			
13	I would like to escape from my thoughts and feelings	IE	.79	.79
	(Ich würde gerne vor meinen Gedanken und Gefühlen flüchten)			
5	I feel powerless to change things	EE	.78	.77
	(Ich fühle mich machtlos, Dinge zu ändern)			
12	I feel powerless to change myself	IE	.75	.78
	(Ich fühle mich machtlos, mich selbst zu ändern)			
15	I would like to get away from who I am and start again	IE	.75	.72
	(Ich wäre gerne nicht mehr ich selbst und möchte nochmals neu beginnen)			
4	I often have the feeling that I would just like to run away	EE	.70	.82
	(Ich habe oft den Wunsch, einfach wegzurennen)			
1	I am in a situation I feel trapped in	EE	.75	.75
	(Ich fühle mich wie gefangen)			
2	I am a strong desire to escape from things in my life	EE	.70	.75
	(Ich habe den starken Wunsch, von gewissen Dingen in meinem Leben Abstand zu nehmen)			
3	I am in a relationship I cannot get out of	EE	.66	.50
	(Ich bin in einer Beziehung, aus der ich nicht hinaus kann)			
8	I would like to get away from other more powerful people in my life	EE	.64	.58
	(Ich würde mich gerne von gewissen Leuten fernhalten, die stärker sind als ich)			
10	I feel trapped by other people	EE	.64	.60
	(Ich fühle mich von anderen Personen gefangen)			
6	I feel trapped by my obligations	EE	.62	.62
	(Ich fühle mich gefangen durch meine Verpflichtungen)			

PP: Paper-Pencil-Sample, OL: Online-Sample, IE: Internal Entrapment, EE: External Entrapment, Subscale: Subscale in the original study of Gilbert and Allan [5].

**Table 4:** Correlation coefficients of entrapment and related constructs.

	Entrapment		
	PP sample (*N* = 170)	OL sample (*N* = 370)	RT sample (*N* = 100)
Depressive symptoms	.62**	.72**	.70**
Stress	.69**	.62**	.75**
Hopelessness	.61**	.50**	.62**

*P < .05. **P < .01, PP: Paper-Pencil-Sample, OL: Online-Sample, RT: Retest-Sample.

Entrapment and depression scores were log transformed.

**Table 5:** Hierarchical regression analyses predicting depressive symptoms (CES-D).

Paper ‘n pencil sample (*N *= 170)								
Model	Predictors	*β*	*T*	*r*	*R* ^2^	*F*(*df*)	Δ*R* ^2^	Δ*F*(*df*)
Step 1								
	Constant		−2.17*					
	Stress	.51	8.73**	.62				
	Hopelessness	.33	5.62**	.50				
					.48	78.09 (2,167)**		

Step 2								
	Constant		.25					
	Stress	.29	4.55**	.61				
	Hopelessness	.17	2.91**	.50				
	Entrapment	.42	6.37**	.72				
					.58	77.88 (3,166)**	.10	40.52 (1,166)**

Online Sample (*N* = 370)								
Model	Predictors	*β*	*T*	*r*	*R* ^2^	*F*(*df*)	Δ*R* ^2^	Δ*F*(*df*)

Step 1								
	Constant		16.87**					
	Stress	.44	9.26**	.59				
	Hopelessness	.28	5.79**	.52				
					.41	124.83 (2,367)**		

Step 2								
	Constant		18.33**					
	Stress	.28	5.08**	.59				
	Hopelessness	.17	3.40**	.52				
	Entrapment	.32	5.57**	.62				
					.45	100.37 (3,366)**	.05	31.02 (1,366)**

Entrapment and depression scores were log-transformed. *P < .05, **P < .01, PP: Paper-Pencil-Sample, OL: Online-Sample.
